# Editorial Comment: Feasibility of Robot - assisted Segmental Ureterectomy and Ureteroureterostomy in Patient with High Medical Comorbidity

**DOI:** 10.1590/S1677-5538.IBJU.2016.0026.1

**Published:** 2017

**Authors:** Alejandro R. Rodriguez

**Affiliations:** 1 Department of Urology and Urology Oncology, Samaritan Medical Center, Watertown, New York, USA

In this video Mello et al. (1), demonstrate the case of a Robot assisted segmental ureterectomy for a patient with a solitary kidney with high medical co-morbidities that had a single localized ureteral lesion consistent with a urothelial neoplasm.

The surgical team carefully diagnosed and staged the ureteral tumor. A formal cystoscopy, bladder washing cytologies, retrograde pyelogram and ureteroscopy was performed to verify that this tumor was localized, single and that there were no other concomitant pathology such as carcinoma in situ of the upper collecting system.

Nephroureterectomy remains the gold standard for the surgical management of upper tract transitional cell carcinoma; However, recently, minimally invasive approaches including endourological (for non invasive disease) as well as laparoscopic or robotic assisted for segmental resections of the distal ureter with ureteral reimplantation has been reported with great oncological and clinical outcomes (for non invasive and invasive disease).

The authors clearly follow oncological principles of resection and anastomosis of clean ureteral margins. They demonstrate the importance of clipping the ureter above and below the tumor with a clear margin of resection. This will prevent spillage of neoplastic cells into the peritoneal cavity, preventing peritoneal carcinomatosis. In this case, the ureters were anastomosed without tension and the patient profitted from a less morbid approach to deal with a single ureteral tumor.

The final pathology revealed a pT3 high grade urothelial carcinoma with associated carcinoma in situ. The prognosis of these cases is poor, but the option of local control in a patient with high medical co-morbidities and a solitary kidney is clearly evident. Finally, we congratulate the authors for such a great demonstration of a Robotic assisted segmental resection of the ureter for a clinically localized ureteral tumor

*Alejandro R. Rodriguez, MD* *Department of Urology and Urology Oncology,* *Director of Robotics and Minimally Invasive Surgery* *Samaritan Medical Center, Watertown, New York, USA* *830 Washington St, Watertown, NY 13601, USA* *Telephone: +1 315 785-4000* *E-mail: armbkdd@yahoo.com*
